# Horizontal gene transfer in an acid mine drainage microbial community

**DOI:** 10.1186/s12864-015-1720-0

**Published:** 2015-07-04

**Authors:** Jiangtao Guo, Qi Wang, Xiaoqi Wang, Fumeng Wang, Jinxian Yao, Huaiqiu Zhu

**Affiliations:** State Key Laboratory for Turbulence and Complex Systems and Department of Biomedical Engineering, College of Engineering, Peking University, Beijing, 100871 China; Center for Quantitative Biology, Peking University, Beijing, 100871 China; School of Life Sciences, Peking University, Beijing, 100871 China

**Keywords:** Environmental microbes, Metagenome, Microevolution, Computational identification

## Abstract

**Background:**

Horizontal gene transfer (HGT) has been widely identified in complete prokaryotic genomes. However, the roles of HGT among members of a microbial community and in evolution remain largely unknown. With the emergence of metagenomics, it is nontrivial to investigate such horizontal flow of genetic materials among members in a microbial community from the natural environment. Because of the lack of suitable methods for metagenomics gene transfer detection, microorganisms from a low-complexity community acid mine drainage (AMD) with near-complete genomes were used to detect possible gene transfer events and suggest the biological significance.

**Results:**

Using the annotation of coding regions by the current tools, a phylogenetic approach, and an approximately unbiased test, we found that HGTs in AMD organisms are not rare, and we predicted 119 putative transferred genes. Among them, 14 HGT events were determined to be transfer events among the AMD members. Further analysis of the 14 transferred genes revealed that the HGT events affected the functional evolution of archaea or bacteria in AMD, and it probably shaped the community structure, such as the dominance of G-plasma in archaea in AMD through HGT.

**Conclusions:**

Our study provides a novel insight into HGT events among microorganisms in natural communities. The interconnectedness between HGT and community evolution is essential to understand microbial community formation and development.

**Electronic supplementary material:**

The online version of this article (doi:10.1186/s12864-015-1720-0) contains supplementary material, which is available to authorized users.

## Background

Horizontal gene transfer (HGT), also known as lateral gene transfer, is defined as the movement of genetic material between phylogenetically unrelated organisms by mechanisms other than parent-to-progeny inheritance. Usually, this horizontal flow of genetic material utilizes three routes: conjugation, transduction, or transformation. Currently, it is widely believed that HGT plays an important role in prokaryotic evolution. Through the acquisition of new genes and functions, the recipient organism can accelerate evolution and adapt to new ecological niches [1, 2]. With the availability of a large number of complete genome sequences, many possible transferred genes were identified in prokaryotes [3, 4]. For example, 755 of 4288 genes were recognized as horizontally transferred into *Escherichia coli* since it diverged from the *Salmonella* lineage about 100 million years ago [5]. Also, the hyperthermophilic bacteria *Thermotoga maritima* is believed to have undergone high rates of genetic exchange with archaeal species sharing its extreme habitat [6]. Overall, it is estimated that 1.6–32.6 % of the genes in microbial genomes have been acquired by HGT [7].

Although HGT has been widely studied in isolated microorganisms, the understanding of HGT events in natural prokaryotic communities is superficial because of the complexity of unculturable characteristics of most microbes on the earth [8, 9]. In fact, many microorganisms live as communities and interact with each other. They exhibit complex social relationships and co-evolve to continuously adapt to the specific environment. As an important driving force of evolution, HGT is thought to significantly influence the dynamics of microbial communities [10, 11]. Therefore, studies of HGT events in a mixture of microbial genomes living in natural communities are worthwhile and are now expected to be realized through the development of metagenomics [12, 13]. With metagenomic and metafunctional genomics data, the developments in microbial communities can be explored at the genome scale using the novel approaches of metagenomics, and it is possible to enhance our understanding of the principles of multi-species consortia and biocomplexity [14]. A recent study reported that the percentage of possible HGT events is close to that of complete genomes in several representative samples, including whale fall, Sargasso sea, farm soil, and human gut [11]. Also, some metagenomic sequencing projects mentioned the possible gene transfer events among various microbial genomes in a specific environment [15–17]. Many viewpoints, which were formed by studying individual prokaryotic genomes, suggested that HGT events contributed to the evolution and adaptation of species [18]. These points can be proposed in microbial communities reflected in metagenome data. For example, HGT events in metagenomes could be driven by factors of both environment and community composition, could accelerate evolution and adaptation to environments, and could happen to some species more than others [11]. However, these points are largely in a state of conjecture because of the challenge of the complexity of sequenced genome collections; therefore, there is an urgent need to confirm and study these HGT in detail.

As mentioned above, the main challenge of analyzing HGT in metagenomes is the complexity of metagenomic sequences, which cannot be circumvented because most of the current sequenced data were recovered directly from environmental samples. To be more specific, an awkward situation, i.e., large numbers of mixed short DNA reads or contigs belonging to many different species (we know little about the population structure for some environmental samples and how many species the communities comprise), made it difficult to analyze genomic sequences and detect HGT in metagenomic sequences using existing computational methods. Fortunately, with the rapid development of metagenome projects, people can now use a few well-studied metagenomic datasets that avoid the above troubles. One example is the dataset of metagenomic sequences in the acid mine drainage (AMD) constructed from studies by the Banfield group [15]. As a typical metagenome, the biofilm growing on the surface of flowing AMD belongs to a low-complexity and relatively self-contained ecosystem [19]. With the advance of high-throughput sequencing strategies, genomes of microorganisms in the AMD community were directly sequenced from the environment. The researchers definitely identified 11 microorganisms with complete or near complete genomic sequences. More importantly, each contig can be exactly traced to its source species. Furthermore, microorganisms in AMD have demonstrated the possibility of gene transfer, and some evidence has been observed. For example, some possible phage genes and integrases with a broad host range were found, and gene transfer through transduction is thus possible among these AMD organisms [15]. With these properties of data, one may apply the computational methods to metagenomic sequence analysis just like it is applied to individual genomes.

In this paper, we used the AMD metagenomic sequences as a model to investigate the putative HGT events that occurred among members within this natural community of microorganisms. We first annotated the protein-coding regions in the metagenomic sequences using the current computational tools. With a phylogenetic approach based on accurate gene reannotation, we detected a set of gene families with a total of 119 genes that showed phylogenetic tree incongruities, implying that they are probably horizontally transferred among genomes in AMD and the nine previously isolated organisms. Among them, 14 HGT events were determined as transfer events among the eight AMD members. The 14 transfer events happened in both directions of gene movement between bacteria and archaea. Further functional and pathway analysis of transferred genes revealed that these HGT events affected the functional evolution of archaea or bacteria in AMD, and they probably shaped the community structure of the AMD ecosystem. With several representative cases via computational analysis of metagenomes in this paper, our exploratory study also presented a supporting view of the role of HGT within community members in the functional distribution and evolution among community members.

## Results

### HGT identification and overview of HGTs among AMD microbial genomes

**Fig. 1:**
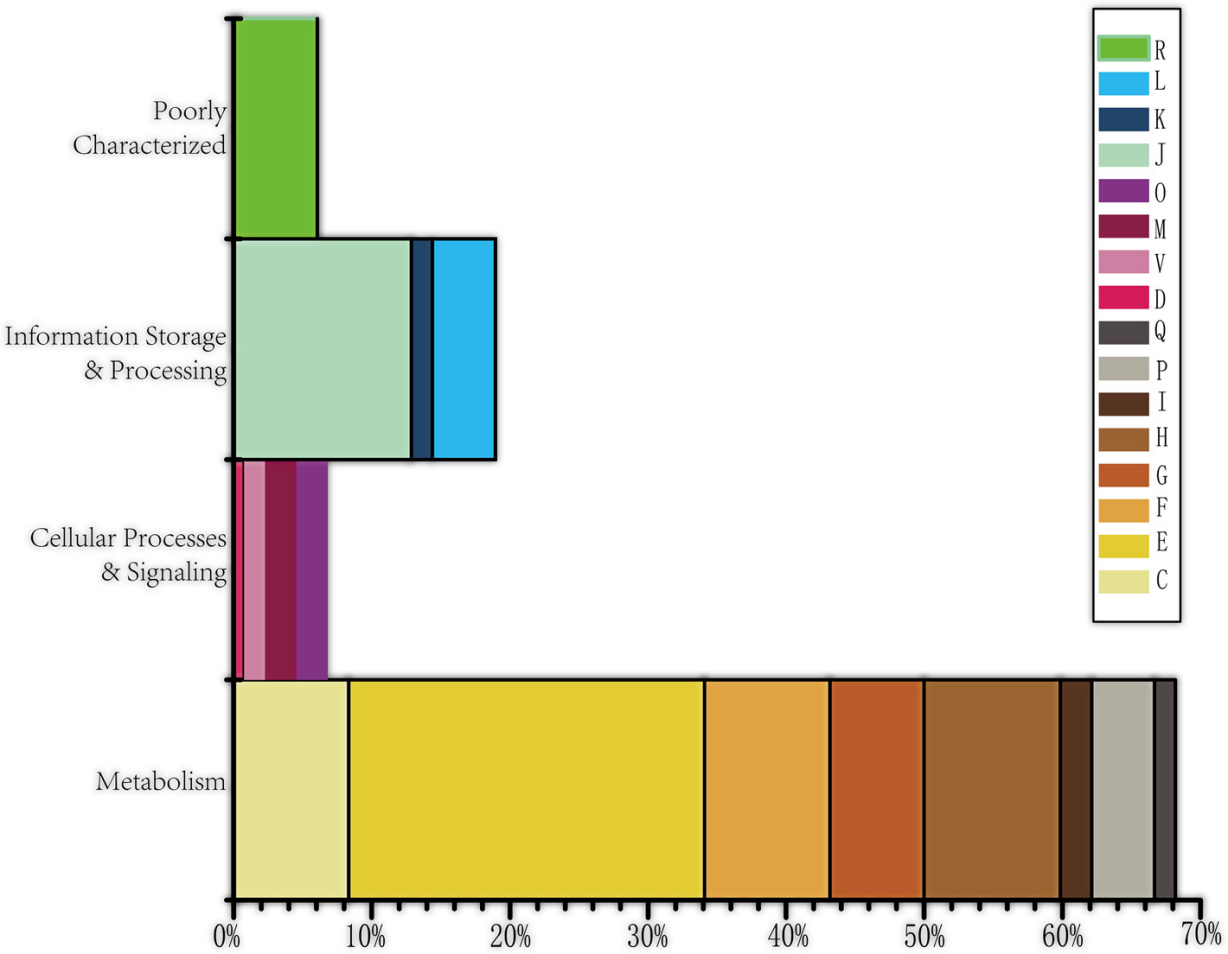
Functional classification of 119 predicted transferred genes. The x-axis represents the frequency of genes in each COG categories. The y-axis shows the four main COG categories, and each colored bar represents a subclass in this category. (Functional categories of subgroups of COG: [J] translation, ribosomal structure, and biogenesis; [A] RNA processing and modification; [K] transcription; [L] replication, recombination, and repair; [B] chromatin structure and dynamics; [D] cell cycle control, cell division, and chromosome partitioning; [Y] nuclear structure; [V] defense mechanisms; [T] signal transduction mechanisms; [M] cell wall/membrane/envelope biogenesis; [N] cell motility; [Z] cytoskeleton; [W] extracellular structures; [U] intracellular trafficking, secretion, and vesicular transport; [O] posttranslational modification, protein turnover, and chaperones; [C] energy production and conversion; [G] carbohydrate transport and metabolism; [E] amino acid transport and metabolism; [F] nucleotide transport and metabolism; [H] coenzyme transport and metabolism; [I] lipid transport and metabolism; [P] inorganic ion transport and metabolism; [Q] secondary metabolite biosynthesis, transport, and catabolism; [R] general function prediction only; and [S] function unknown)

We first reported our identification of the genes that were horizontally transferred among the genomes including eight organisms in AMD and nine previously isolated organisms. Using the method of strict reciprocal best basic local alignment search tool (BLAST) hit [20, 21] based on the accurate gene reannotation by the current computational tools [22] or annotation from the National Center for Biotechnology Information (NCBI) [23], we obtained 251 orthologous gene families that were present in more than seven genomes in which at least one genome was from AMD, while 185 of them showed phylogenetic tree incongruities using an approximately unbiased test (AU test) [24]. Furthermore, 66 of the 185 phylogenetic trees were excluded because the differences in the branch length were not the same as those of the organism tree, which were probably caused by different evolution rates and could not represent HGT events. Therefore, 119 genes [Additional file 1] with significant evolution incongruence were identified in the study. Of these 119 genes, most of them demonstrate a function related to metabolism, especially amino acid transport and metabolism (Fig. 1). In fact, the metabolic network of *E. coli* has a lot of changes and growth to acquire various external nutrients in the past 100 million years caused by HGT instead of gene duplication [25]. In this case, over 66 % of transferred metabolism-related genes can build a metabolically flexible lifestyle to facilitate the environmental adaptation in this extreme acidic and metal-rich environment [26]. Therefore, it is reasonable to hypothesize that the 119 genes indicate possible horizontal transfer events among genomes in AMD and the nine previously isolated organisms.

We further analyzed the transfer events with a clear evolutionary path in which only AMD organisms were involved. To this end, after a rigorous manual selection from the 119 possible transfer events, 14 genes were determined to be HGT among the eight AMD organisms (Table 1). Furthermore, possibly involved genomes among the eight AMD organisms, the possible donor and recipient organisms, and functions of the 14 genes were identified and listed in Table 1. It is interesting that genetic exchanges of these genes occur between bacteria and archaea in both directions in this community. We noted that two transfer events from bacteria to archaea, which included 11 of the 14 genes, are crucial to the development of the AMD community. This makes a lot of sense because two iron-oxidizing chemoautotrophic bacteria, *Leptospirillum ferrodiazotrophum* and *Leptospirillum rubarum*, dominate this relatively self-contained ecosystem [19]. With respect to the transfer events in this direction, functions of the involved genes affect the AMD microbial community. In the next two subsections, the details of the putative HGT events in this direction are reported. Herein it is noteworthy that the transfer events in the other direction, i.e., from archaea to bacteria, also suggested the significance of transferred genes to the recipients. For instance, two genes, *MesJ* and *Mdh*, were horizontally transferred from archaea to bacteria *L. ferrodiazotrophum* and *L. rubarum*. The MesJ protein is a well-known cell-cycle protein that is directly responsible for lysidine formation and thus is essential for decoding AUA codons *in vivo* [27]. *Mdh*, which was proved to be horizontally transferred in a previous study [28], plays a key role in the cell during growth on methanol. The *CcmA* gene, which was transferred from archaea to the bacterium *L. ferrodiazotrophum*, is the first gene of an eight protein-encoded operon involved in cytochrome c maturation and heme delivery [29]. The evolution of cytochrome c domains has been reported to involve gene transfer events [30]. *CcmA* was identified as the essential gene of the cytochrome c ATPase; therefore, *CcmA* is likely to be transferred to assist the electron transfer process.

**Table 1:** Fourteen horizontally transferred genes with high reliability among eight organisms in acid mine drainage

Gene	Function	Donor and recipient for the possible transfer events
*mesJ*	tRNA(Ile)-lysidine synthase *MesJ*	Archaea to *L. ferrodiazotrophum* and *L. rubarum*
*ccmA*	ABC-type multidrug transport system, ATPase component	Archaea to *L. ferrodiazotrophum*
*gadB*	Glutamate decarboxylase and related PLP-dependent proteins	Bacteria to *F. fer1* and *F. fer2*
*mdh*	Malate/lactate dehydrogenases	Archaea to *L. ferrodiazotrophum* and *L. rubarum*
*metC*	Cystathionine beta-lyases/cystathionine gamma-synthases	Bacteria to *E-plasma,* and *I-plasma*
*rplD*	Ribosomal protein L4	Bacteria to *G-plasma*
*rplE*	Ribosomal protein L5	Bacteria to *G-plasma*
*rplP*	Ribosomal protein L16/L10E	Bacteria to *G-plasma*
*rplR*	Ribosomal protein L18	Bacteria to *G-plasma*
*rplX*	Ribosomal protein L24	Bacteria to *G-plasma*
*rpsC*	Ribosomal protein S3	Bacteria to *G-plasma*
*rpsD*	Ribosomal protein S4 and related proteins	Bacteria to *G-plasma*
*rpsE*	Ribosomal protein S5	Bacteria to *G-plasma*
*rpsJ*	Ribosomal protein S10	Bacteria to *G-plasma*

In this study, we focused on the 14 horizontally transferred genes that could be traced in the AMD evolutionary process; other genes also have the possibility of horizontal transfer among the AMD community members. HGT events are widely involved in microbial genomes in natural communities; however, the computational identification of HGT in genomic sequences remains very difficult in practice [11]. We could have identified more HGT events among the AMD metagenomes. However, to more thoroughly investigate the impacts of HGTs, we selected the genes that were most likely transferred and made a large contribution to the AMD community. A second notable aspect of this study is that we aimed to examine HGT events between members within the microbial community. To be sure, novel genes of a recipient genome were recognized as being transferred by a donor species either from an external environment or internal community. However, as a relatively self-contained ecosystem in an extreme environment, the AMD microorganisms can demonstrate the social behaviors of both cooperation and competition. In this light, we argued that the movement of genetic materials among organisms within the community should be essential to their performance as a whole. Finally, it is certain that the gene transfer events studied in this work were determined by the strict standard of HGT identification adopted here, which also implies that these putative HGT events were identified amongst distantly related species. HGT might be easier between closely related species for which the barriers to the transfer more easily can be surmounted [11]. However, because of the environmental challenges, some gene transfer events would greatly benefit some species or the entire community, leading to a better adaptation to the environment, even with the effort for the exchange of genetic material between distantly related species. In other words, the HGT events reported in this manuscript should be regarded as being nontrivial and essential to the AMD community because of the strong selective pressure and the harsh environment in AMD.

### Acquisition of antibiotic resistance in *G-plasma* via HGT associated with ribosomal proteins

Among the 14 horizontally transferred genes, nine were transferred from bacteria to the archaeon *G-plasma*. Moreover, they encoded ribosomal proteins and the related subunits. Following, we report our detection and analysis of the consequence of these transferred genes on *G-plasma* and the AMD microbial community.

**Fig. 2:**
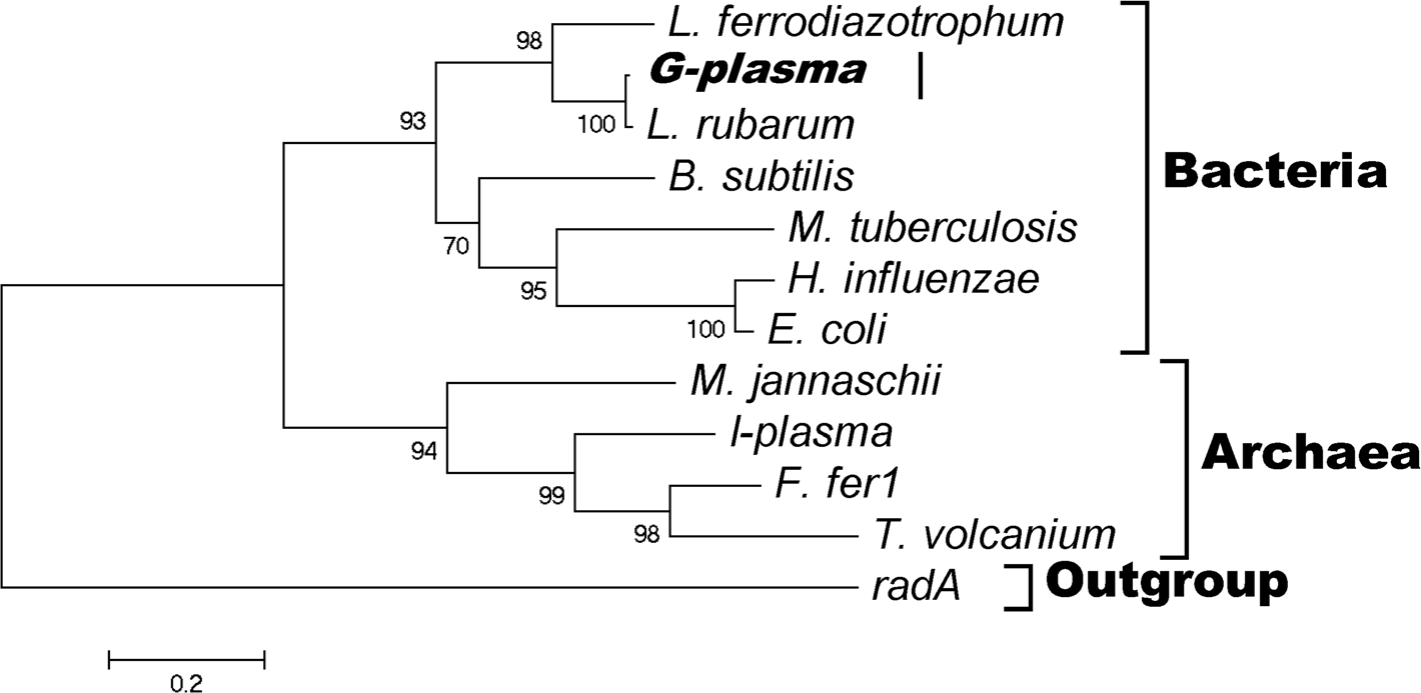
Phylogenetic tree of the *rpsE* gene family. As a conserved gene, *radA* is used as an outgroup. The *rpsE* gene of *G-plasma* is located in a cluster of bacteria, indicating possible horizontal transfer

Ribosomal proteins are widely distributed in prokaryotes, and they are usually involved in information processing (e.g., replication, transcription, and translation) or central metabolism. Genes for ribosomal proteins are generally considered to be housekeeping genes and relatively recalcitrant to HGT; because of this, their sequences are routinely used as phylogenetic markers [31]. However, ribosomal genes have shown a remarkable possibility of being transferred in many prokaryotic genomes [3]. For example, a phylogenetic study on ribosomal protein S14 revealed an unexpected tree topology that was explained by horizontal transfer [32]. Also, ribosomal proteins L32 and L33 have phylogenetic incongruities that resulted from gene transfer [33]. In the current study, one of the evident horizontal transfer events was that of ribosomal protein genes. In *L. ferrodiazotrophum*, we found a contig (gi: 251772484) containing 10 ribosomal genes: *rpsJ*, *rplD*, *rpsC*, *rplP*, *rplX*, *rplE*, *rplR*, *rpsE*, *rpsK*, and *rpsD*. Of these genes, *rpsJ*, *rplD*, *rpsC*, and *rplP* are usually clustered as operon S10, while *rplX*, *rplE*, *rplR*, and *rpsE* are usually clustered as operon *spc*. All of these ribosomal genes are in the positive strand of the contig [Additional file 2: Figure S1], and their protein name and function are listed in Table 2. Looking into the phylogenetic tree of these ribosomal genes, most of them (except *RpsK*) show obvious disagreement with the species tree, strongly indicating possible horizontal transfer events. To be specific, the ribosomal genes of *G-plasma* often have a close relationship with those of *L. ferrodiazotrophum* and *L. rubarum* instead of other plasma species, and they are placed in a cluster of bacteria rather than archaea in the gene tree. The same pattern holds for the ribosomal genes of both the *spc* operon (*rpsE* in Fig. 2, others in Additional file 2: Figures S2–S4) and S10 operon [Additional file 2: Figures S5–S8]. Therefore, ribosomal genes of *G-plasma* show a high possibility of being transferred from bacteria. Considering the close relationship and the same habitat as *Leptospirillum*, it makes sense that *L. ferrodiazotrophum* and *L. rubarum* are the donor of these ribosomal genes for the archaeon *G-plasma*.

**Table 2:** Ribosomal gene information

Protein	Aa	Gene	Operon	SwissProt AC	Function besides Ribosomal Protein
S10	103	*rpsJ*	S10	P02364	-
L4	201	*rplD*	S10	P02388	Macrolide antibiotic resistance
S3	232	*rpsC*	S10	P02352	Form mRNA entry pore
L16	136	*rplP*	S10	P02414	Antibiotic resistance
L24	103	*rplX*	*spc*	P02425	Assembly initiation
L5	178	*rplE*	*spc*	P02389	-
L18	117	*rplR*	*spc*	P02419	-
S5	166	*rpsE*	*spc*	P02356	Antibiotic resistance
S11	128	*rpsK*	*α*	P02366	-
S4	203	*rpsD*	*α*	P02354	Ensure precision of decoding

Notably, these genes were identified as transferred clusters, including two known operons, from bacteria to archaea as shown in Additional file 2: Figure S1. This feature is clearly consistent with the theory of a selfish operon, in which operons are viewed as mobile genetic entities that are constantly disseminated via HGT, although their retention could be favored by the advantage of the co-regulation of functionally linked genes [34]. As a result, functional information for these transferred genes may be inferred by comparing them with the related or analogous genes. An earlier study focusing on the horizontal transfer of *rps14*, which is also a member of the *spc* operon, led to the conclusion that antibiotic resistance can be conferred because *rps14* is known to be involved in antibiotic resistance through the binding of puromycin besides its major role in the assembly of ribosomal 30S subunits [32, 35]. In the current study, although *rps14* does not appear in both *G-plasma* and the two *Leptospirillum* bacteria, we noted that *rpsE*, which is in both *G-plasma* and *Leptospirillum*, has a function that is similar to that of *rps14*, i.e., antibiotic resistance. Therefore, we may safely conclude that *G-plasma* can acquire this function through horizontally transferred *spc* operon genes. In addition, the S10 operon has two genes, *rplP* and *rplD*, that are involved in antibiotic resistance. These observations led to the explanation that *G-plasma* acquired these genes for antibiotic resistance. As predicted by the selfish operon model [34], these transferred gene clusters should allow cells in the AMD environment to demonstrate the metabolic benefits of antibiotic resistance; moreover, they can enhance the fitness of *G-plasma* as the recipient species.

Another question of interest is how the transferred genes associated with antibiotic resistance influence the microbial community in AMD. We may just as well learn this point from their role in *L. ferrodiazotrophum* and *L. rubarum*, the two most dominant organisms in AMD. The amount of antibiotic resistance proteins in *L. rubarum* was correlated with the growth stage of biofilms [36]. In early growth stage samples, *L. rubarum* dominates the community, and its repressors of antibiotic resistance genes are abundant. With the growth of biofilms, increasing types of microorganisms compete for limited resources (e.g., nitrogen, oxygen, and phosphate), forcing *L. rubarum* to reinforce its competitiveness and self-protection. A previous study demonstrated that antibiotic resistance proteins from *L. rubarum* were more abundant during late-stage growth [36]. Clearly, antibiotic resistance is closely related to the population of individual species in the natural microbial community. It is interesting to note that *G-plasma* is the dominant archaeon in AMD. Based on proteomic data across 28 microbial community samples, *G-plasma* constituted about 9.0 ± 4.9 % of the community [37]. Deduced by analogy, *G-plasma* and other archaea should also encounter a similar situation, and the archaeon with a competing advantage will survive easier than archaea without the advantage. As shown by the population distribution, *G-plasma* is no doubt a strong competitor. The horizontal acquisition of antibiotic resistance genes is a possible advantage that protects and enhances its competitiveness, making *G-plasma* the largest group among AMD archaea. As a result, both these genes and their horizontally transferred relatives that provide antibiotic resistance influence the growth of species and shape the population structure of the AMD microbial community.

Taken together, the transferred gene clusters of ribosomal genes are associated with antibiotic resistance, leading to the fitness improvement of the archaeon *G-plasma* and the community structure in AMD. It should be pointed out that the requirement of antibiotic resistance, as the selective pressure, may force microorganisms to overcome the possible difficulties of horizontal transfer because these horizontally acquired antibiotic resistance genes are of great importance in the life of *G-plasma* in the toxic environment of the AMD, and they may be vital in making *G-plasma* the largest group of AMD archaea.

### Possible impact of *gadBC* operon transfer on acid resistance in *Ferroplasma*

In this subsection, we discuss the putative horizontal transfer of the *gadB* gene of the *gadBC* operon from bacteria to two archaea of the *Ferroplasma* genus. The *gadBC* operon corresponds to the function of acid resistance. Acid resistance is perceived as an essential property of microorganisms living in AMD. The glutamate decarboxylase (GAD) system, which is common in bacteria and some eukaryotic genomes, has been extensively studied for its major role in acid resistance in organisms such as in *E. coli*, *Shigella flexneri*, and *Listeria monocytogenes* [38–41]. An essential role of biochemical pathways is to yield cell–cell messengers [41]. The GAD system is usually composed of three genes, *gadA*, *gadB*, and *gadC*. The *gadA* and *gadB* genes usually have high sequence similarity and encode two biochemically indistinguishable glutamate decarboxylases, and the *gadC* gene encodes a glutamate/GABA antiporter. The *gadB* and *gadC* genes are organized into a functionally important operon, *gadBC* [42]. By producing alkaline γ-aminobutyrate and utilizing an intracellular proton, functions that involve the *gadBC* operon, cells can adapt to low pH [43].

**Fig. 3:**
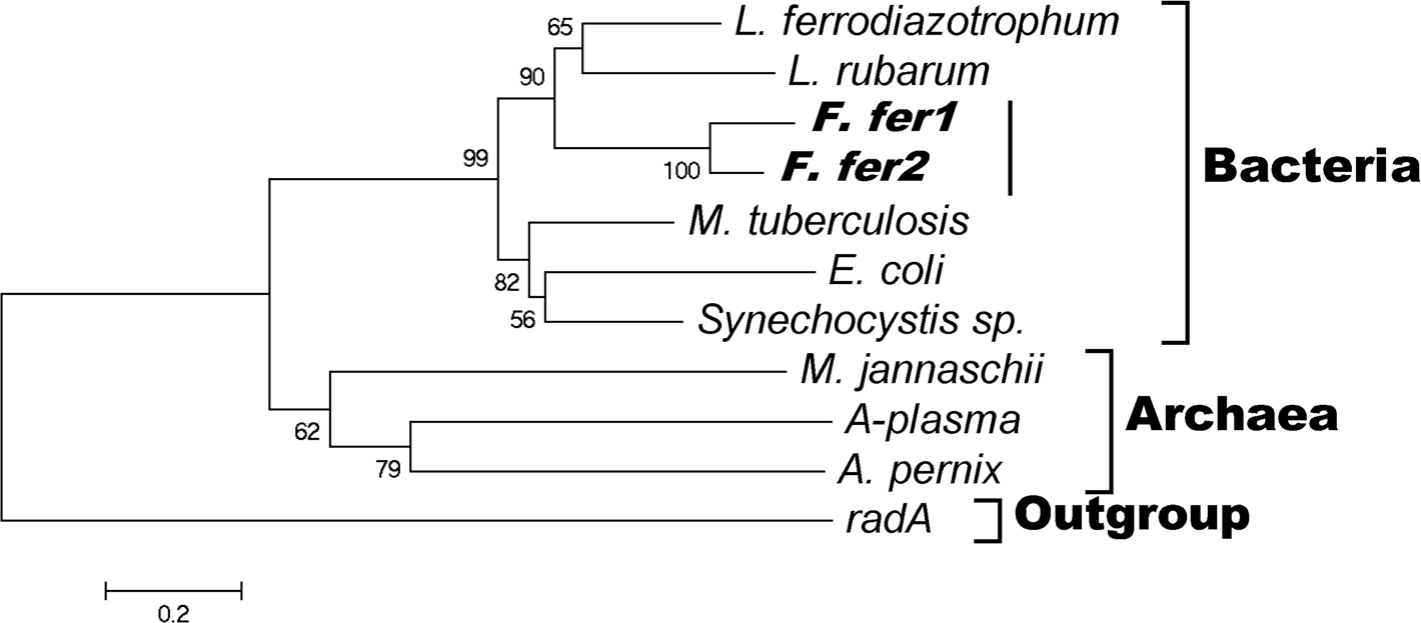
Phylogenetic tree of the *gadB* gene family. As a conserved gene, *radA* is used as an outgroup. The *gadB* genes of *Ferroplasma fer1* and *Ferroplasma fer2* are located in a cluster of bacteria, indicating possible horizontal transfer

Through investigation of the phylogenetic tree of *gadB*, we found that there was an obvious incompatibility with the organism tree, and *gadB* from *Ferroplasma fer1* and *Ferroplasma fer2* were misplaced within the cluster of bacteria; the closest relatives were genes from *L. ferrodiazotrophum* and *L. rubarum* (Fig. 3). This incompatibility indicates a possible transfer event. Moreover, transposase genes were found in the neighborhood of the *gadBC* operon in both *F. fer1* and *F. fer2*, providing additional evidence for the HGT events [44]. Because of the low conservation and poor annotation quality of the *gadC* gene in public databases, it is difficult to estimate the true representation of *gadB* in most microbial genomes even with examples of its potential role in a few well-known organisms such as *E. coli* and *S. flexneri*. Although the *gadC* gene in both *F. fer1* and *F. fer2* is located close to that of *L. ferrodiazotrophum* and *L. rubarum* in the phylogenetic tree, we did not analyze its confused and incongruous evolution in the current study. However, because there was only one transcription start site for the *gadBC* operon regardless of the inducing condition [45] and no obvious promoter could be found upstream of *gadC*, we cannot exclude the possibility of the same transfer event including the *gadC* gene, leading to the transfer of both the *gadB* and *gadC* gene. Furthermore, the prediction of 13 transmembrane passes with TMHMM 2.0 [46] inferred the integral function of *gadC* in *Ferroplasma*. An ancestor of *Ferroplasma* originally acquired the *gadB* gene by horizontal transfer (transferred as the *gadBC* operon), and *L. ferrodiazotrophum* and *L. rubarum* might be the donors because of their close relationship and presence in the same habitat in which they overcame the acid environment to use more resources.

On the basis of the function of the GAD system, it is reasonable to suggest that the acquisition of the *gadBC* operon allowed *F. fer1* and *F. fer2* to better resist the extreme acid environment. Although there are other mechanisms to resist a low-pH environment [47], the horizontally transferred *gadBC* operon plays an important role in the efficient utilization of a proton by *F. fer1* and *F. fer2* to resist the extreme acidic environment. Combined with the putative transfer events of the *spc* operon and S10 operon from bacteria to *G-plasma*, there seems to be a tendency toward the movement of genes with operon structures from bacteria to archaea. In general, this agrees with the selfish operon hypothesis [34]. However, the two cases described in this report indicate that most transferred genes involved in the same operon have a closely functional relationship. Moreover, they play an essential role in the requirements of antibiotic or acid resistance in recipient archaea. This differs from the viewpoint that the majority of genes in transferred operons are nonessential genes with related functions [34, 48]. In an environment that seems so harsh, organisms have to retain their beneficial genetic resources to survive the external stresses of the environment.

## Discussion

As an important driving force of the evolution of microorganisms, HGT has been extensively studied in prokaryotes. However, the theories of HGT of natural microorganisms in extreme environments remain open; currently, these theories seem to be largely in a state of conjecture because of the challenges associated with the complexity of sequenced metagenomes. In this paper, we used AMD metagenomic sequences as a model metagenome, which avoided the difficulties of HGT identification in metagenomic data from natural environmental samples. The strategy designed to identify HGT events is based on a strict standard in the current study. Because phylogenetic reconstruction was assumed to cause inherent noise and possible artifacts, we used an AU test, which is regarded as an appropriate approach to evaluate the phylogenetic topology to yield a discriminating result and dramatically reduce the noise of phylogenies. Herein, we attempted to focus on a set of putative HGT events that occurred among members within the AMD microbial community. As we have seen with our computational identification strategy, apparent HGT events occurred among the microorganisms in AMD. Our investigation thus provided insight into understanding a potential role of HGT in the environmental microbial communities. Although there are possible limitations of computational identification of HGT events, the bioinformatics strategy avoids the experimental difficulties and the challenges associated with the complexity of multiple species in a natural niche. Furthermore, because the methodology of computational identification has been widely used in genomics research, studies based on these methods give researchers important clues to explore HGT events and genomic evolution [49–51].

Through gene transfer, functional traits were introduced into the recipient microorganisms, and those that overcame selective pressure are presented by heredity. For example, antibiotic resistance, transferred via the HGT of ribosomal genes in AMD, allowed microorganisms to grow in the presence of certain poisonous compounds. Also, with the acquisition of acid resistance, such as that via HGT of the *gadBC* operon, cells could remove intracellular protons and adapt to the acidic environment. Therefore, these transferred genes may greatly contribute to the metabolic and physiological requirements of the extreme environment and provide microorganisms with innovative functional solutions to adverse environmental problems. Aside from the impact on the recipients, the influence of HGT also influenced the structure of the entire community or environment. As mentioned above, transfer events of ribosomal genes enhanced the competence of *G-plasma* and greatly influenced the community structure in AMD. Previous studies demonstrated that gene transfer may promote cooperation among microorganisms in some situations and was correlated with biofilm formation [52]. It should also be noted that the extreme environment and community could influence the possibility of HGT. For example, the acid solution prevents gene transfer by the uptake of naked DNA in AMD. Consequently, gene transfers were definitely influenced by both the natural environment and community members. In summary, our findings, which are based on bioinformatics analysis, revealed that gene transfer events potentially contribute functional innovation to the recipients, as well as further shape the dynamic evolution and population structure of the special microbial community.

Furthermore, from an evolutionary standpoint, it is reasonable to discuss HGT in the context of the community instead of single microorganisms to understand the social relationships and dynamics of community members in a natural environment. It has been pointed out that communities are ideal candidates for genetic exchange among microorganisms [53]. In comparison, single microorganism had low competitive competence; therefore, living as consortia could benefit many members in the community through events such as the acquisition of new genes by HGT. Especially in biofilms, which are microbial cells in densely packed communities, the possibility of HGT is very high [52]. Eventually, HGT in a community will accelerate single microorganism evolution and adaptation to the environment, and the subsequent evolution of social relationships will also be influenced. Therefore, trends of gene transfer and community dynamics are highly correlated. Research on the interconnectedness between HGT and community evolution will expand our view of the impacts on the recipient, community, and environment.

At present, the clustering property of transferred genetic materials remains uncertain and is therefore an interesting problem for HGT studies. In the current study, our two detailed cases clearly exemplified this issue: they were all connected with operon structure, especially the large gene clusters in the ribosomal gene transfer. As we mentioned above, the selfish operon model argued that operon organization allowed efficient horizontal transfer of genes that were otherwise susceptible to loss by genetic drift [34]. Moreover, recent research showed that the amount of genetic material that can be moved horizontally may include small gene fragments, entire operons, superoperons that encode complex biochemical pathways, and whole chromosomes [54]. Thus, it is not surprising that our two cases were identified as transfer events at the operon level. However, compared with transfer events of a single gene, to what extent did HGT events involve gene clusters? If genes were transferred by cluster structure, what intrinsic factor caused it? These questions are largely unsolved mysteries of genomic evolution. The current study provides nontrivial support to extend the research to natural community microorganisms. A further problem is how these transferred genes were accepted and how they retained their function in the recipient genome. Whether these newly acquired genes can survive in the recipient genome depends on the evolution of their sequence characteristics, transcription, and expression mechanisms. To this end, we analyzed the genes associated with ribosomal proteins that were transferred from bacteria to *G-plasma*. We examined the sequence pattern of regions upstream of the transferred ribosomal genes in the recipient genome. As a result, the translational and transcriptional signals in the upstream region were much like those of *G-plasma* instead of those of the donor bacteria. This is compatible with the fact that phylogenetic methods were used to detect ancient transfer events. It is uncertain whether this appearance was caused by the convergent evolution of the originally transferred signals, or if these genes used the signals from the recipient genome. A recent study demonstrated that operons were often acquired with their regulators to facilitate the evolution of the transferred genes [55]. This is in accord with the speculation that *gadB* and *gadC* of the *gadBC* operon might have been transferred together and used one transcription signal that was located upstream of *gadB*. In this case, the convergent evolution of transferred genes with their original translation regulatory regions seems likely. However, it is clear that further investigation will be required to reach a full understanding of this problem.

## Conclusions

Using the AMD metagenomic data as a model metagenome, our exploratory study provided unique insight into HGT events among microorganisms in natural communities. It is important to discuss all possible contributions of HGT among community members to an individual species, the entire microbial ecosystem including its dynamic evolution, and the related environment. In this regard, our analyses partly address these outstanding questions and shed light on functional metagenomics and social evolution in microbes from a metagenomic perspective. This study will stimulate future investigation of these topics propelled by an interdisciplinary exchange between microbiologists, evolutionary biologists, and bioinformaticians.

## Methods

### Obtaining sequences and gene annotation

Complete or near-complete sequences of 11 organisms were constructed from three samples in AMD [15, 56] and downloaded from NCBI [23] (http://www.ncbi.nlm.nih.gov/). Three *ARMAN* organisms were not considered in this study because of their small genome size and gene number. Recently, several tools to predict coding regions in metagenomic sequences were developed [20, 21, 52]. The eight remaining organisms (including two bacteria and six archaea), were analyzed using MetaGeneAnnotator [57] to identify genes and MetaTISA [22] to locate Translation Initiation Sites (TISs). Besides the AMD microorganisms, nine previously isolated organisms (five bacteria and four archaea) were added to the phylogenetic analysis to obtain improved resolution. Protein sequences of these nine isolated organisms were downloaded from NCBI to allow orthologous gene family identification. Information about these organisms is listed in Table 3.

**Table 3:** General features of genomes in the study

Genome	Kingdom	Size (Mb)	GC (%)	#Gene
*E. coli*	Bacteria	4.64	51	4145
*B. subtilis*	Bacteria	4.22	44	4176
*M. tuberculosis*	Bacteria	4.41	66	3988
*H. influenzae*	Bacteria	1.91	38	1792
*L. rubarum*	Bacteria	2.64	55	1640
*L. ferrodiazotrophum*	Bacteria	2.82	58	2747
*Synechocystis PCC 6803*	Bacteria	3.95	47	3179
*A-plasma*	Archaea	1.94	46	2202
*G-plasma*	Archaea	1.78	38	2405
*E-plasma*	Archaea	1.58	38	1600
*I-plasma*	Archaea	1.69	44	1735
*F. fer1*	Archaea	1.46	36	1526
*F. fer2*	Archaea	1.82	37	1424
*A. pernix*	Archaea	1.67	56	1700
*T. volcanium*	Archaea	1.58	40	1501
*M. jannaschii*	Archaea	1.66	31	1714
*S. solfataricus*	Archaea	2.99	36	2978

### Phylogenetic tree construction

To construct the gene family phylogenetic tree, the strict reciprocal best BLAST hit method [20, 21] with E-value cutoff 10^−5^, identity >25 %, and alignment length >60 % was used to identify orthologous gene families. These gene families were aligned with ClustalW 2.0 [58] using default parameters. For each gene family, a maximum likelihood tree was built using the proml program in the PHYLIP package [59]. Each dataset was replicated 100 times, and the last consensus tree was decided by majority rule with Consense in the PHYLIP package [59].

**Fig. 4:**
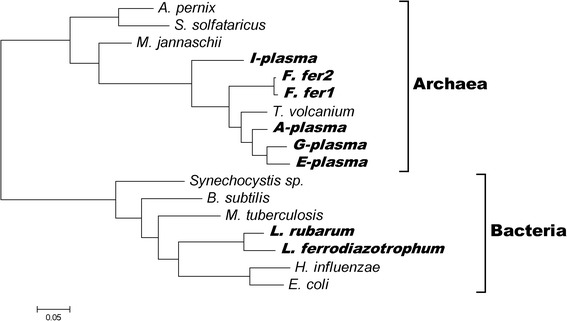
Phylogenetic tree of the 16S rRNA from genomes in this study. Microorganisms from acid mine drainage are shown in bold

The organism tree was constructed using 16S rRNA as a phylogenetic marker (Fig. 4). The corresponding 16S rRNA sequences of AMD organisms were predicted using RNAmmer [60], and those of other genomes were downloaded from RDP database (Ribosomal Database Project) [61]. Accordingly, a maximum likelihood tree of these organisms was built with MEGA 5.0 [62].

### AU test

Usually, the incompatibility of orthologous gene family phylogenetic trees and organism phylogenetic trees indicate the possibility of transfer events. Herein, the AU test [24], a powerful method to infer gene transfers as tested on *in silico* data [51], was used to assess the confidence of tree selection. Log-likelihoods of phylogenetic trees were estimated using Codeml from the PAML package [63] with the Dayhoff substitution matrix. Then, the *p*-value of the AU test was calculated using CONSEL [64]. If the *p*-value was less than 0.05, the hypothesis that the orthologous gene family evolved according to the organism tree was rejected; i.e., a possible horizontal gene transfer might have occurred.

## Additional files

Additional file 1:
**Lists the information of 119 predicted transferred genes.**
Additional file 2:
**Lists all proposed horizontally transferred genes, the phylogenetic trees that are not included in the article, and positions of ribosomal genes in the** ***Leptospirillum ferrodiazotrophum*** **and** ***G-plasma*** **genomes.**

## Abbreviations

HGT: Horizontal gene transfer
AMD: acid mine drainage
AU: approximately unbiased
GAD: glutamate decarboxylase
COG: Cluster of Orthologous Groups of proteins
