# Supplementary material for: Horizontal gene transfer in an acid mine drainage microbial community
Source: BMC Genomics. 2015 Jul 4;16(1):496. doi: 10.1186/s12864-015-1720-0 (PMC4490635; doi:10.1186/s12864-015-1720-0)

Figure S1: Positions of ribosomal genes on *L. ferrodiazotrophum* and *G-plasma*. They locate on the same contig in *L. ferrodiazotrophum*, while in *G-plasma* they are on four different contigs.

Figure S2: Phylogenetic tree of *RplX* gene family.

Figure S3: Phylogenetic tree of *RplE* gene family.

Figure S4: Phylogenetic tree of *RplR* gene family.

Figure S5: Phylogenetic tree of *RpsJ* gene family.

Figure S6: Phylogenetic tree of *RplD* gene family.

Figure S7: Phylogenetic tree of *RpsC* gene family.

Figure S8: Phylogenetic tree of *RplP* gene family.

Figure S1: Positions of ribosomal proteins on *L. ferrodiazotrophum* and *G-plasma*.

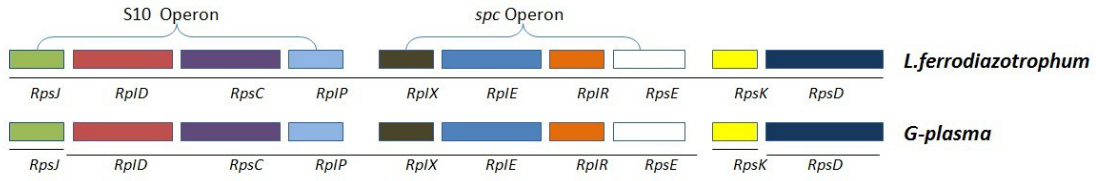

Figure S2: Phylogenetic tree of *RplX* gene family.

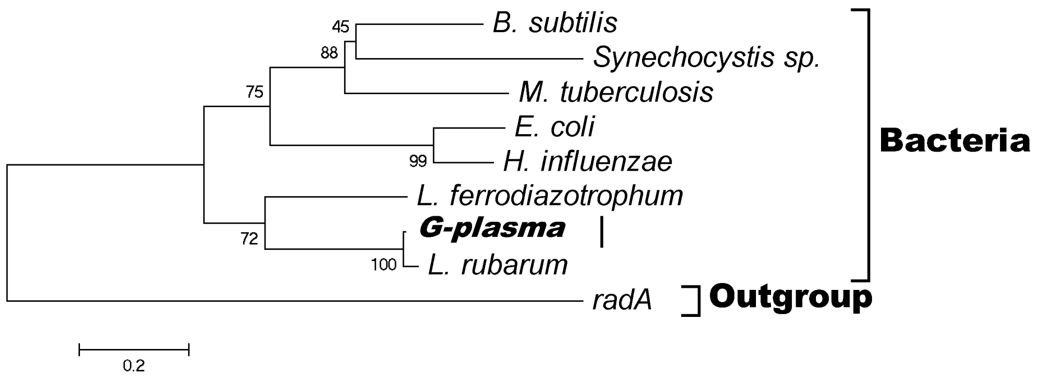

Figure S3: Phylogenetic tree of *RplE* gene family.

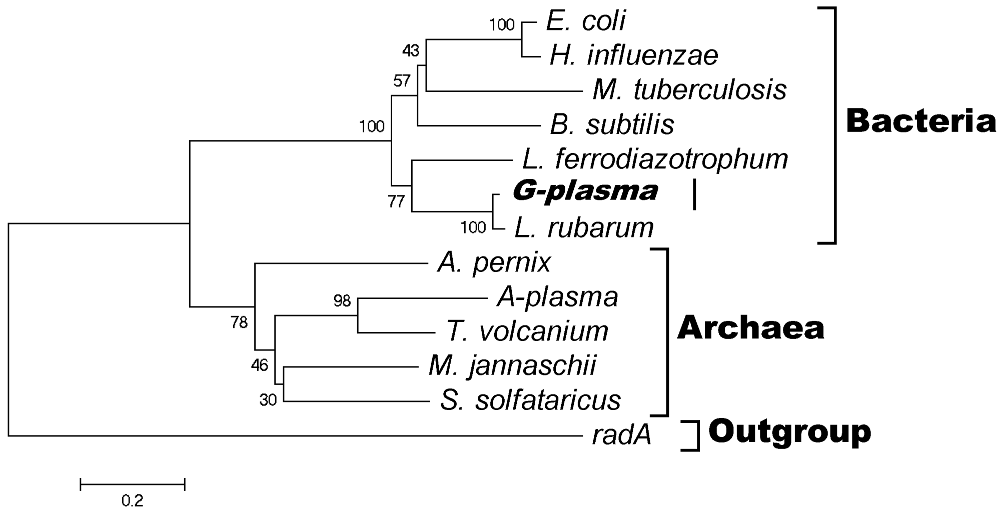

Figure S4: Phylogenetic tree of *RplR* gene family.

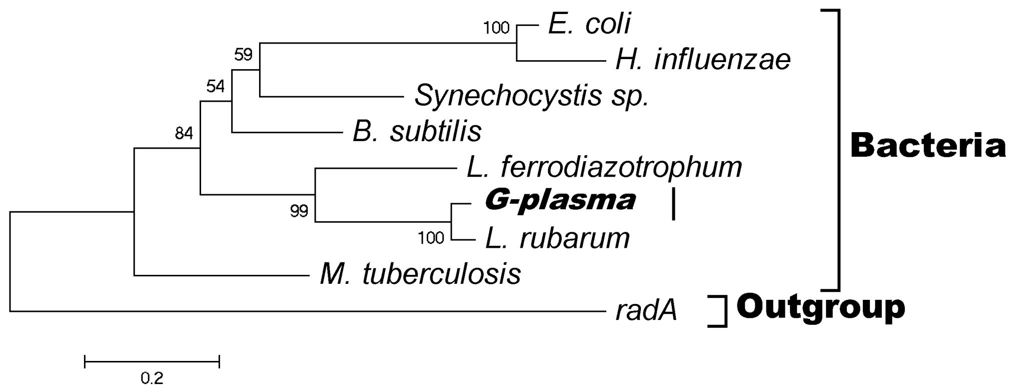

Figure S5: Phylogenetic tree of *RpsJ* gene family.

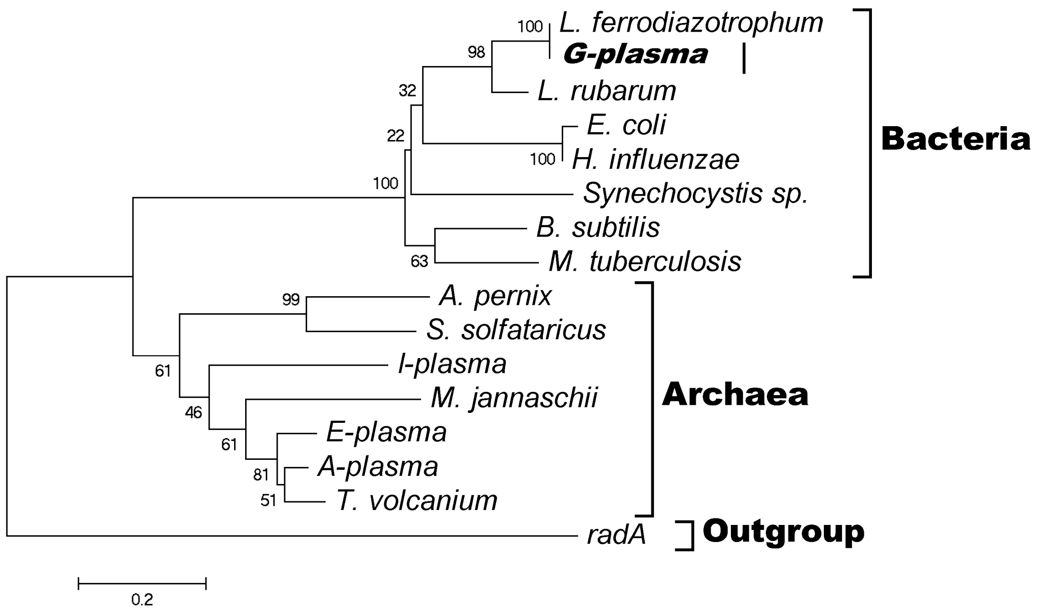

Figure S6: Phylogenetic tree of *RplD* gene family.

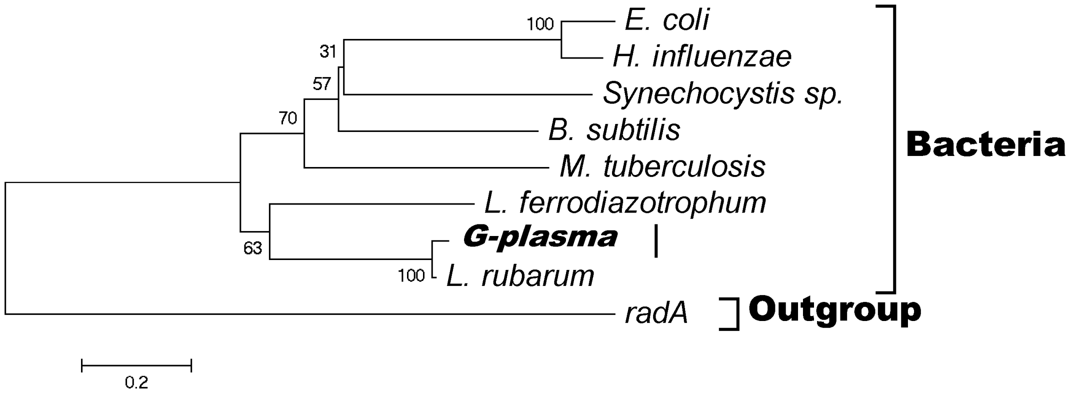

Figure S7: Phylogenetic tree of *RpsC* gene family.

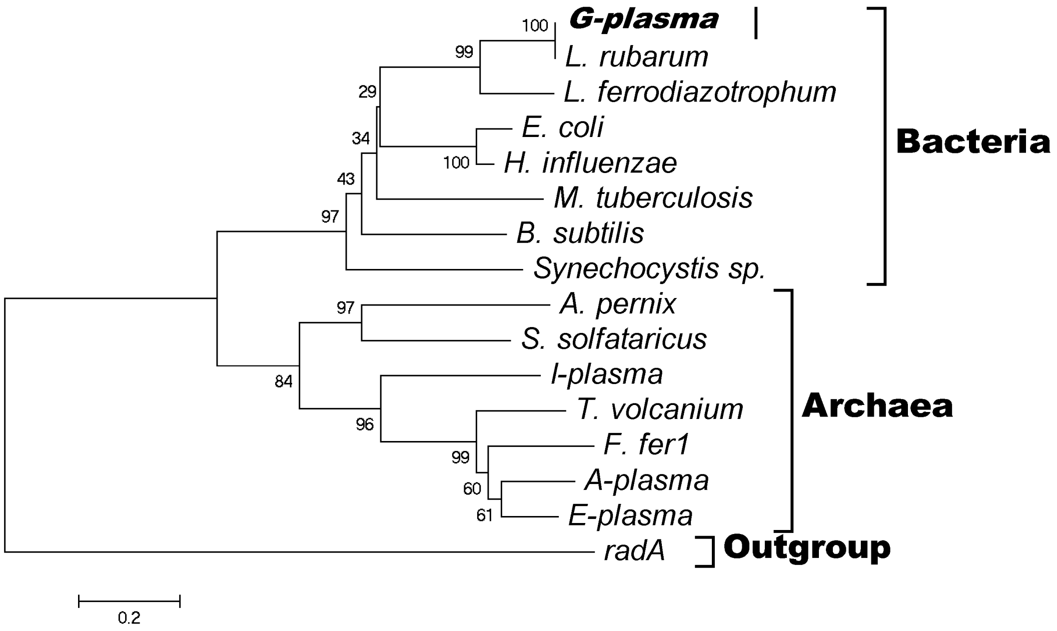

Figure S8: Phylogenetic tree of *RplP* gene family.

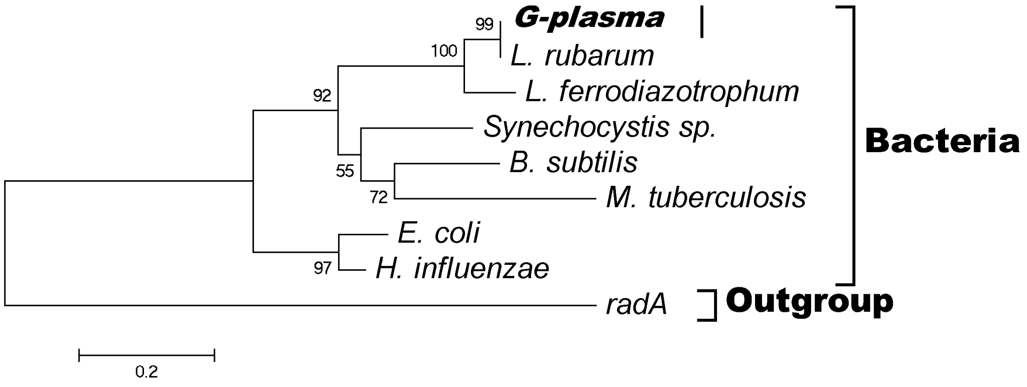

Supplement: Additional file 2: — Lists all proposed horizontally transferred genes, the phylogenetic trees that are not included in the article, and positions of ribosomal genes in the Leptospirillum ferrodiazotrophum and G-plasma genomes. [file 12864_2015_1720_MOESM2_ESM.pdf]
